# Supplementary figures and images for: Potent universal beta-coronavirus therapeutic activity mediated by direct respiratory administration of a Spike S2 domain-specific human neutralizing monoclonal antibody
Source: PLoS Pathog. 2022 Jul 21;18(7):e1010691. doi: 10.1371/journal.ppat.1010691 (PMC9302814; doi:10.1371/journal.ppat.1010691)

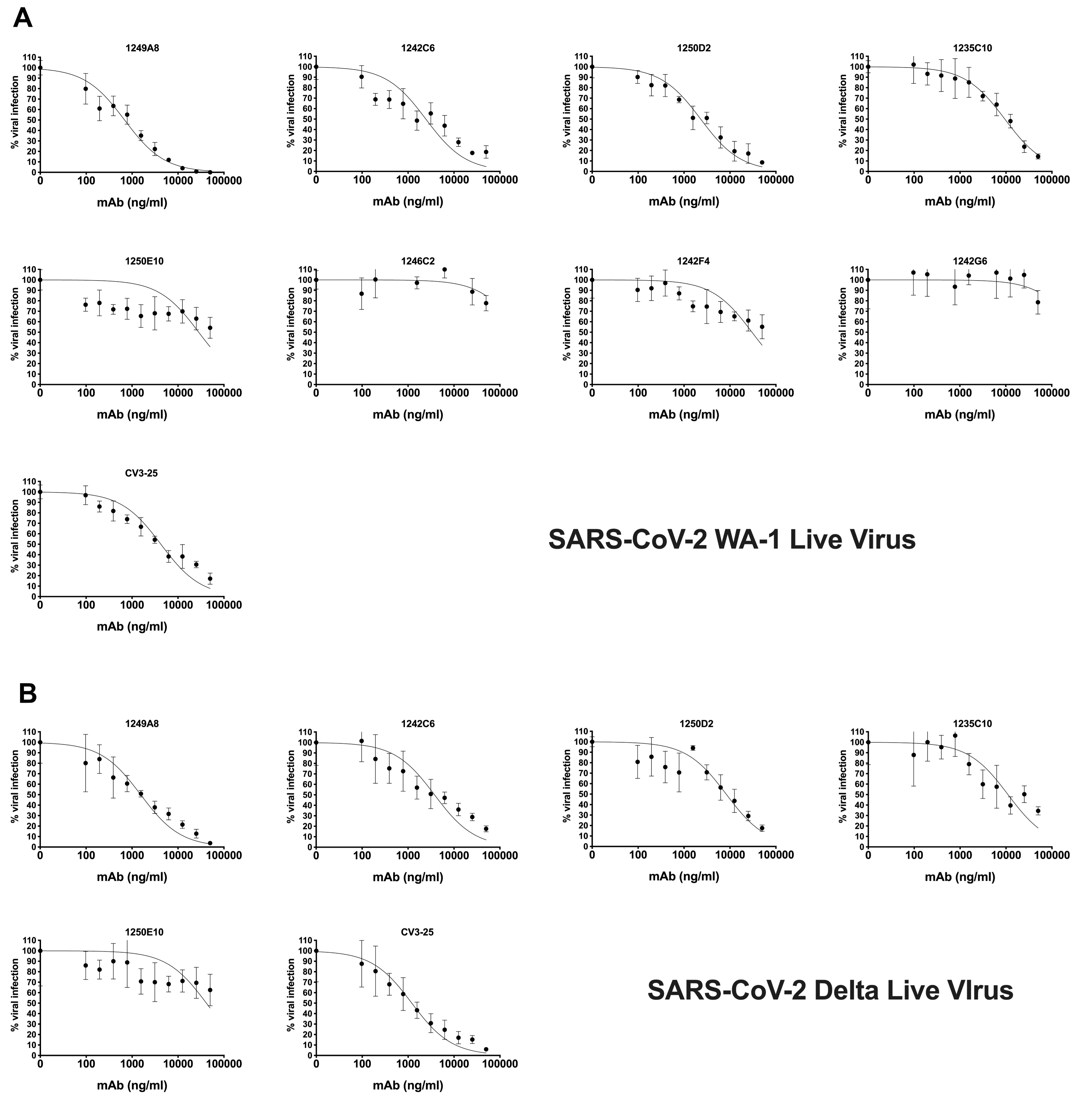

Supplement: S1 Fig — Vero E6 cells were infected with SARS-CoV-2 WA-1 (A) or SARS-CoV-2 Delta (B), and 1 hour after viral adsorption mAb was added at indicated concentrations in triplicate. At 24 h p.i. cells were fixed, stained with anti-NP mAb 1C7C7 and quantified using ELISPOT. (TIF) [file ppat.1010691.s001.tif]

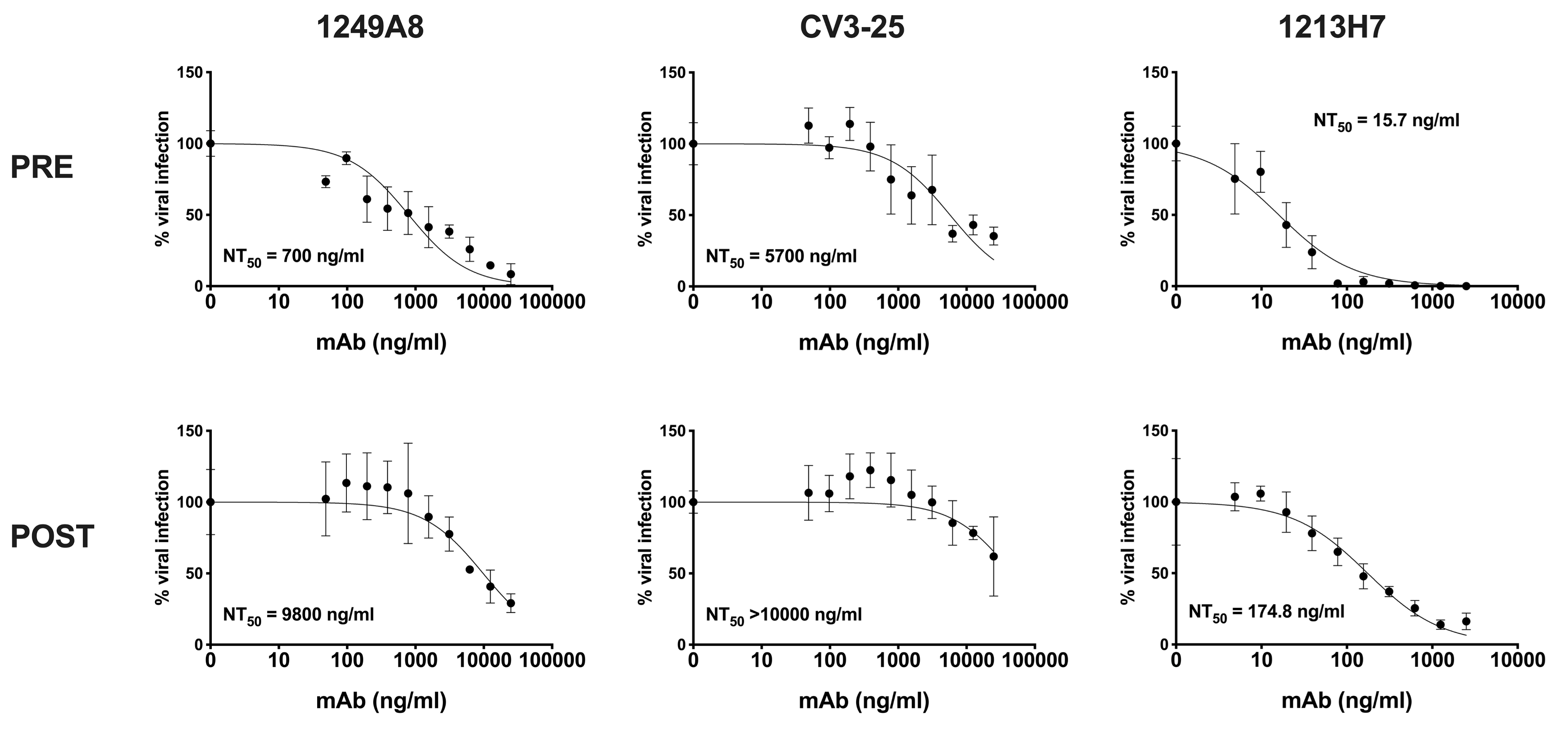

Supplement: S2 Fig — For pre-treatment (top), 100 PFU/well of SARS-CoV-2 WA-1 containing indicated concentrations of mAbs were mixed and incubated for 1h. Vero HL cells were infected with virus-mAb mixture as virus adsorption for 1h, followed by changing media. For post-treatment (bottom), Vero HL cells were infected with 100 PFU/well of SARS-CoV-2 WA-1. After 1 h of viral adsorption, the media was changed with indicated concentrations of mAb. At 24 h p.i., infected cells were fixed, immunostained using anti-NP mAb 1C7C7, and quantified using ELISPOT. (TIF) [file ppat.1010691.s002.tif]

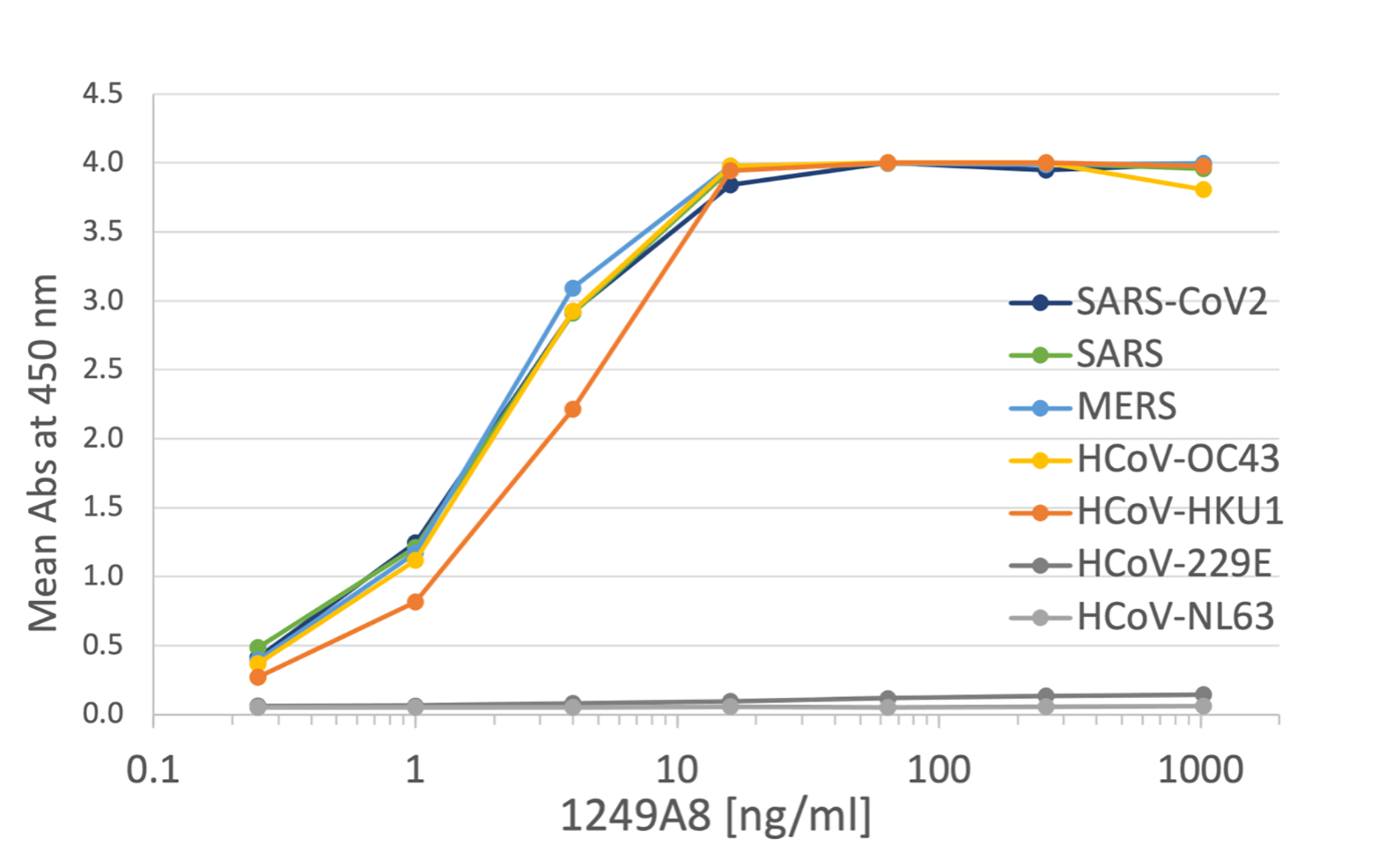

Supplement: S3 Fig — 1249A8 was tested at indicated concentrations by ELISA for binding to indicated Spike proteins. (TIF) [file ppat.1010691.s003.tif]

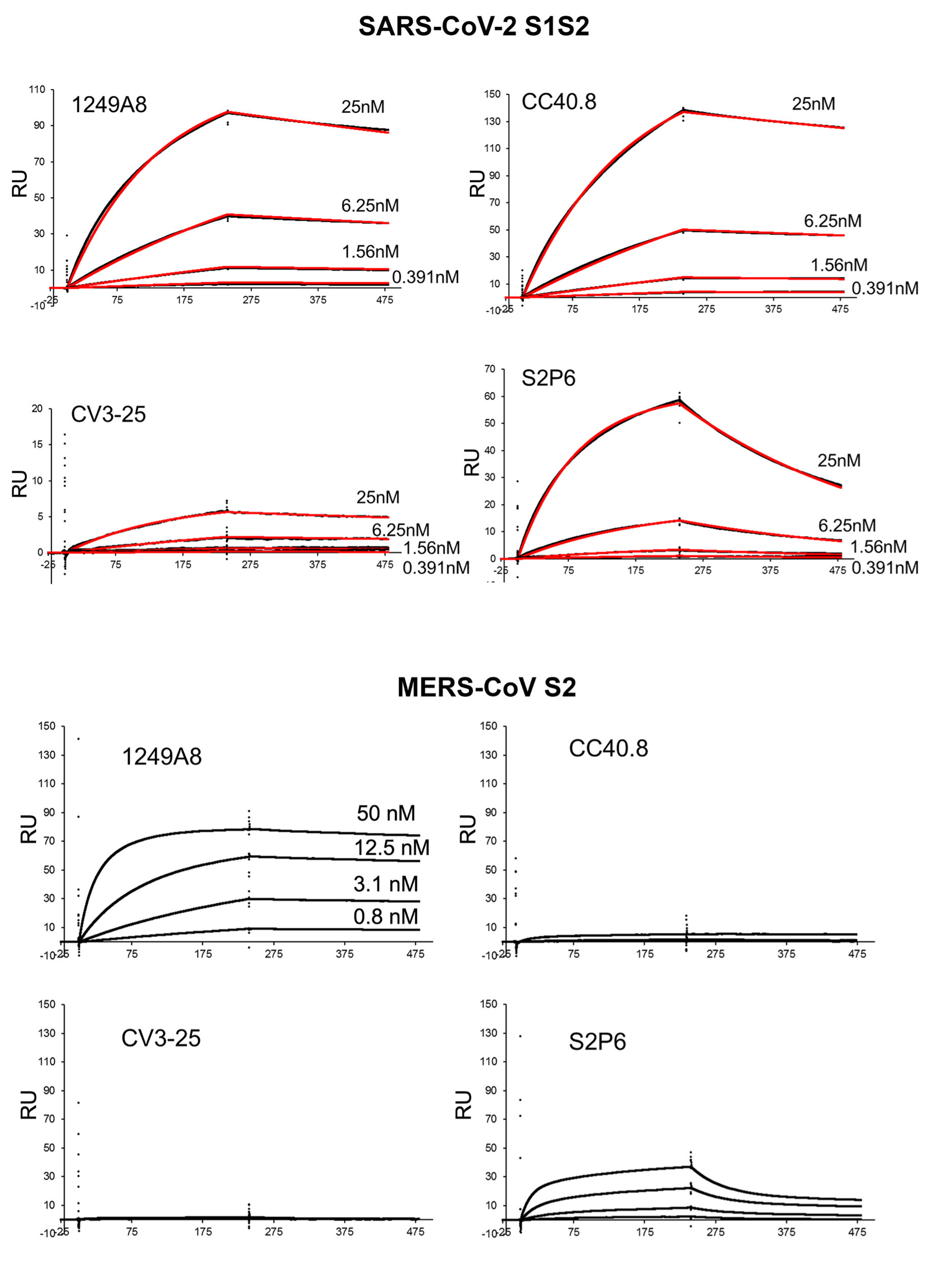

Supplement: S4 Fig — Using a Biacore T200, mAbs were immobilized and the binding kinetics for the interaction between mAbs and Spike protein was determined by injecting four concentrations of SARS-CoV-2 S1S2 (top) or MERS-CoV S2 (bottom). (TIF) [file ppat.1010691.s004.tif]

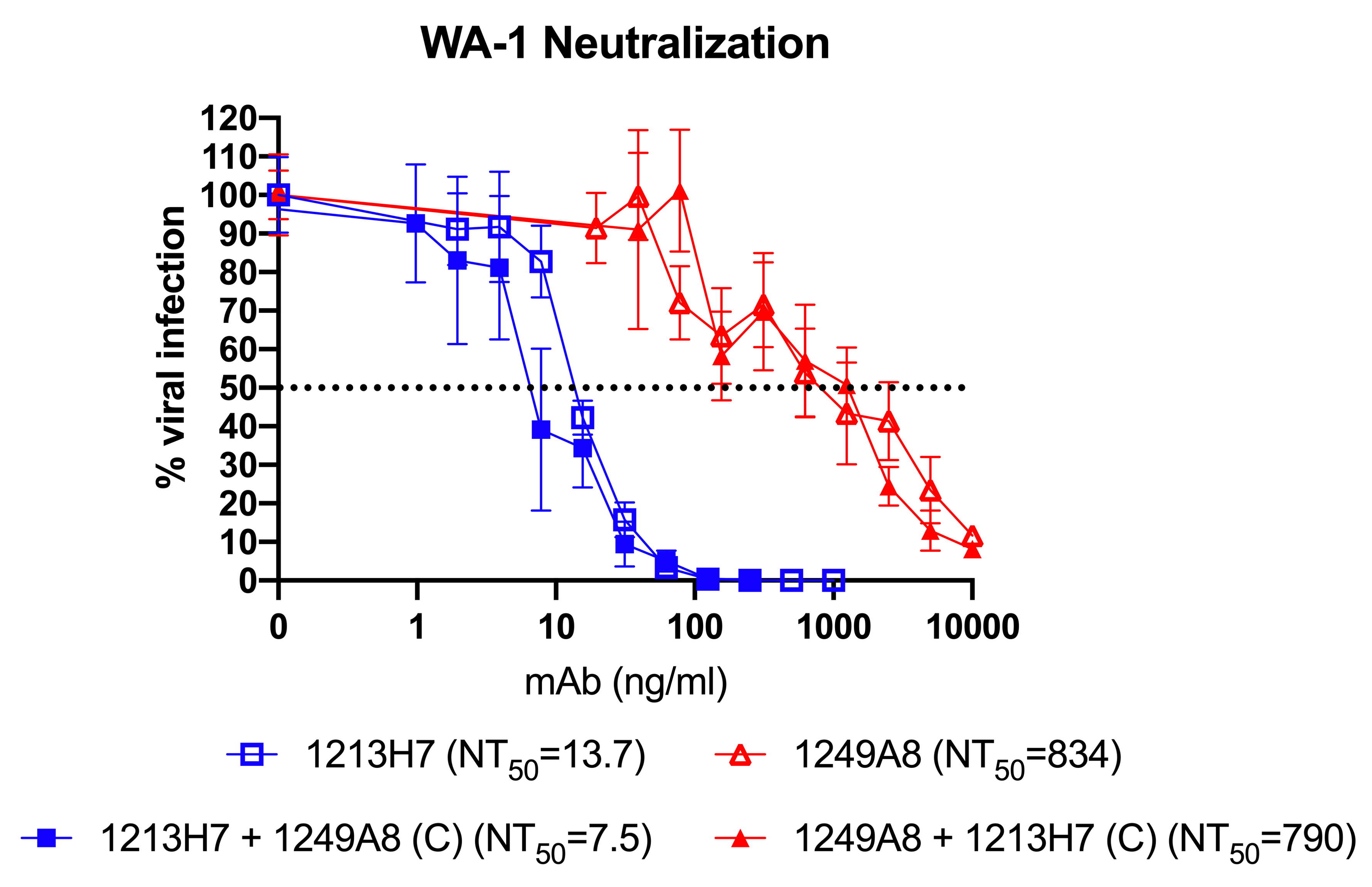

Supplement: S5 Fig — Vero AT cells were infected with SARS-CoV-2 WA-1 and after 1 h of viral adsorption, the indicated mAb(s) was added and at 24 h.p.i infected cells were fixed for virus titration by immunostaining assay. 1213H7 and 1249A8 were tested alone (open symbols) and together keeping 1213H7 constant (C) (50 ng/ml) or 1249A8 constant (2 μg/ml) and titrating the reciprocal mAb (closed symbols). Resulting NT50 (ng/ml) are indicated. (TIF) [file ppat.1010691.s005.tif]
